# Identifying In-Hospital Risk Factors for Post-liver Transplant Seizures

**DOI:** 10.7759/cureus.78233

**Published:** 2025-01-30

**Authors:** Franco E Appiani, Jesica A Pszenyckyj, Lucas Muller, Carla Bolaño, Guido Vazquez, Blas Couto, Carlos S Claverie, Alfredo Thomson

**Affiliations:** 1 Department of Neurology, Hospital CIMA Sanitas, Barcelona, ESP; 2 Institute of Neurosciences, Favaloro Foundation University Hospital, Buenos Aires, ARG; 3 Department of Neurology, John Walton Muscular Dystrophy Research Centre, Newcastle Upon Tyne, GBR

**Keywords:** liver transplantation, neurological complications, perioperative risk factors, postoperative seizures, predictive modeling

## Abstract

Background

Liver transplantation (LT) is a life-saving intervention for end-stage liver disease; however, postoperative complications, particularly neurological issues such as seizures, pose significant challenges. This study aims to identify perioperative factors associated with seizures following LT and develop a predictive risk model. By recognizing these in-hospital risk factors, clinicians may tailor perioperative management to mitigate seizure risk and improve neurological outcomes.

Methodology

We conducted a retrospective observational study of adult patients who underwent LT at a tertiary referral center between January 2009 and January 2019. Data were collected for the perioperative period, spanning seven days pre-LT to 30 days post-LT. Variables included demographic, clinical, neurological, and liver-related data. Statistical analyses compared patients with and without seizures using appropriate tests for categorical and continuous variables. A predictive model for seizures was developed using in-hospital factors. It was then internally validated and evaluated for accuracy, sensitivity, specificity, and receiver operating characteristic curve. From this model, a practical clinical risk score was created.

Results

Of 376 patients, 40 (10.6%) experienced seizures within 30 days post-LT. The median age was 54.8 years, and 41% were males. Chronic liver failure was the primary indication for LT, with alcohol abuse, hepatitis C, and hepatitis B being the most common etiologies. Patients’ preoperative conditions included hepatocellular carcinoma (23%), chronic hyponatremia (22.6%), and prior kidney failure (22.1%). Portosystemic encephalopathy (PSE) was present in 46.5% of patients. Patients’ preoperative blood tests revealed low hemoglobin (mean: 10.8 g/dL, SD: 2.2), hyponatremia (mean: 134 mEq/L, SD: 5), elevated international normalized ratio (mean: 2.21, SD: 1.56), high bilirubin (mean: 8.74 mg/dL, SD: 10.23), and creatinine (mean: 1.41 mg/dL, SD: 1.95). Combined liver and kidney transplantation was performed in 9.2% of cases, while 15.4% were emergency procedures. Graft complications occurred in 25% of patients, with functional delay being the most frequent (14.5%). Immunosuppressive regimens included prednisone with tacrolimus or mycophenolate. Seizures typically occurred on post-LT day seven (interquartile range: 5-13) and included uncertain onset (40%), non-convulsive status epilepticus (30%), generalized seizures (22.5%), convulsive status epilepticus (5%), and focal seizures (2.5%). Treatment was administered to 85% of seizure patients, primarily with levetiracetam (75%). Using seven acute perioperative variables, i.e., age at transplantation, history of PSE, pre-LT epilepsy diagnosis, pre-LT hemoglobin, procedure duration, graft cold ischemia time, and intraoperative blood transfusion, we developed a risk score for post-LT seizures. This score achieved an accuracy of 0.72 (95% CI: 0.63-0.80) and an area under the curve (AUC) of 0.90.

Conclusions

This study identifies key in-hospital factors associated with seizures following LT and presents a predictive risk model based on clinical preoperative and surgical variables. With an AUC of 0.90, the model demonstrates strong discriminative ability, suggesting it is a robust tool for predicting seizures in the immediate post-LT period. Further prospective multicenter studies are needed to externally validate the model and risk score, thereby enhancing its clinical applicability.

## Introduction

Technological advancements have positioned transplantation as the leading treatment for end-stage organ failure, with a marked increase in its adoption in developing countries over recent years. In particular, liver transplantation (LT) has seen significant growth, with a 40.8% increase in the past decade, now ranking as the second most common transplant procedure [1-3].

LT serves as a vital intervention for patients with advanced chronic liver disease or acute liver failure, offering the potential for improved survival and quality of life. However, the procedure is not without risks, as immediate post-LT complications continue to pose significant challenges, contributing to substantial morbidity and mortality. Neurological complications, including encephalopathy, epileptic seizures, and stroke, are observed in up to 49% of LT cases and represent a leading cause of disability in this patient population [4-13]. Risk factors such as portosystemic encephalopathy (PSE), infections, and severe ascites have been identified as contributors to these neurological outcomes [4,5,13].

Specifically, the occurrence of seizures following LT warrants careful attention due to its multifactorial etiology involving preoperative conditions, intraoperative factors, and postoperative complications. Seizures not only increase the risk of mortality by up to tenfold but may also result in long-term cognitive impairment, depending on the underlying mechanism and severity [14-17].

In this context, identifying perioperative and in-hospital risk factors for seizures holds significant potential for improving patient care. By recognizing individuals at higher risk, clinicians can implement targeted preventive measures, such as more frequent neurological monitoring, earlier diagnostic imaging, optimized immunosuppressive regimens, or prophylactic antiseizure therapies (ASMs) ultimately aiming to reduce seizure incidence, shorten hospital stays, and improve long-term neurological and graft outcomes.

Therefore, the objectives of this study are twofold: (1) to describe the clinical and neurological characteristics of a cohort of LT patients, focusing on those who develop seizures; and (2) to develop a predictive model and risk score based on clinically relevant perioperative variables. We hypothesize that an evidence-based stratification of seizure risk will enhance perioperative decision-making, guide targeted interventions, and improve neurological and overall transplant outcomes in this vulnerable population.

## Materials and methods

Design and population

We conducted a retrospective observational study of adult patients who underwent LT at a single tertiary referral center between January 2009 and January 2019. All data were retrieved from comprehensive digital medical records (DMRs), which routinely document preoperative, intraoperative, and postoperative clinical variables for transplant recipients.

Inclusion and exclusion criteria

Inclusion criteria consisted of adult patients (≥18 years) who underwent any form of LT and those with complete DMR data spanning seven days before LT through 30 days after LT. Exclusion criteria encompassed patients lacking key data for accurate seizure classification and individuals undergoing multiple LT procedures in the same admission, where only the final hospitalization was considered. A seven-day pre-LT to 30-day post-LT window was selected to encompass both immediate preoperative risk factors (such as metabolic derangements) and early postoperative complications (such as graft dysfunction and infections), which prior studies have identified as critical determinants of neurological outcomes [4,5].

Dataset and group definition

Demographic, clinical, neurological, and liver-related variables were extracted from DMRs. All seizure diagnoses and classifications were confirmed by board-certified neurologists, considering the International League Against Epilepsy (ILAE) criteria [17,18]. Non-convulsive status epilepticus (NCSE) and convulsive status epilepticus (CSE) were considered in the seizure group. Patients included in the seizure group also considered an acute symptomatic seizure, secondary to acute central nervous system insult (stroke, infection, or trauma) or a significant electrolyte/metabolic disturbance (serum Na <125 or >155 mmol/L; serum glucose <60 or >600 mg/dL; total calcium <8.5 or >11 mg/dL; serum magnesium <1.2 mg/dL; ammonia >100 µmol/L; urea nitrogen >100 mg/dL; drug/alcohol withdrawal) according to the ILAE criteria. Bedside prolonged electroencephalograms (EEGs) were performed whenever a clinical suspicion of seizure activity arose, in accordance with the American Clinical Neurophysiology Society guidelines. CT was the first-line imaging for acute neurological change, and MRI was ordered if CT findings were inconclusive. Seizures that occurred from postoperative day 0 through day 30 were attributed to the LT procedure. Patients with a documented seizure within seven days before LT were considered to have a preoperative seizure history, distinguishing them from patients who first experienced seizures post-LT. In patients who experienced seizures, clinical and laboratory data were collected within 24 hours of the event. This included laboratory tests to identify severe metabolic triggers, in-hospital EEG recordings, and neuroimaging studies. Additional data included seizure classification based on the ILAE criteria, ASM administration, and recurrence rates [18]. Mortality rates were analyzed at 30 days and one year post-LT.

Data analysis

All data analyses were conducted in R version 2023.06.2. We began by evaluating normality for continuous variables using the Kolmogorov-Smirnov test or Shapiro-Wilk test. Quantitative variables with normal distributions were summarized using means and standard deviations (SDs), while non-parametric variables were described using medians and interquartile ranges (IQRs). Qualitative variables were reported as frequencies and percentages. Parametric data were compared using the t-test, and non-parametric data were compared using the Mann-Whitney U test or the Kruskal-Wallis test. Categorical data were examined using Fisher’s exact test or chi-square tests.

Data engineering for the predictive model

Predictive variables were preprocessed by selecting relevant predictors. We imputed missing values using median (for numerical) and mode (for categorical) methods when there was low (<10%) missingness. For variables with >10% missingness, a sensitivity analysis comparing models with and without these variables was performed. If a variable’s omission did not adversely affect model performance, it was excluded. We eliminated non-clinically plausible outliers using the quartile method, and constant variables according to their variance. Highly correlated variables >0.9 were excluded, and multicollinearity was addressed by removing predictors with a variance inflation factor >5. The dataset was split into training (70%) and test (30%) subsets. To balance class distributions in the training set, the synthetic minority over-sampling technique was applied, achieving a 1:1 class ratio.

A random forest model was used because of its robust handling of complex interactions and relatively high performance in small-to-medium datasets. It was trained using variables selected for their importance (using stepwise selection) and clinical relevance in the post-surgical context. The model’s performance was evaluated through accuracy, sensitivity, specificity, and area under the curve (AUC) metrics, supplemented by receiver operating characteristic (ROC) curve analysis. We then derived a simplified risk score from the most important random forest predictors. Cutoff thresholds were established using the Youden’s Index and guided by clinical plausibility. Risk categories were developed to facilitate practical bedside application.

## Results

Over a 10-year period, DMRs from 376 patients who underwent LT were analyzed. Among these, 40 (10.6%) patients experienced seizures within 30 days post-LT. The cohort consisted of 41% (155) males, with a median age of 54.8 years at the time of transplantation. The mean in-hospital stay before LT was 3.5 days, with most patients (71.8%, 270) admitted on the day of the procedure. The mean total in-hospital stay was 19.9 days. Mortality was 6.9% (26) at 30 days which increased to 12.8% (48) at one year, with a follow-up rate of 92.8% (349).

The majority of patients (87.2%, 328) suffered from chronic liver failure, primarily due to alcohol abuse (28%, 108), hepatitis C (22%, 86), and hepatitis B (13%, 50). Other notable preoperative conditions included hepatocellular carcinoma (23.7%, 89), chronic hyponatremia (22.6%, 85), and prior kidney failure (22.1%, 83). A history of PSE was observed in 46.5% (175) of patients. In active PSE at transplantation (31.9%, 120), the following grades based on the West Haven classification were recorded: 40% (48) grade 1, 35.8% (43) grade 2, 12.5% (15) grade 3, and 11.7% (14) grade 4. Further baseline characteristics and comparative data between patients with and without seizures are detailed in Table 1.

**Table 1:** Characteristics of liver transplant recipients with and without seizure after surgery. LT: liver transplantation; MDRD: modification of diet in renal disease; MELD: Model for End-stage Liver Disease; RIN: international normalized ratio; PSE: portosystemic encephalopathy

Variable	Overall (n = 376)	No seizure group (n = 336, 89.4%)	Seizure group (n = 40, 10.6%)	P-value
Demographics				
Male, n (%)	155 (41.2)	139 (41.4)	16 (40.0)	NA
Age at transplantation (mean ± SD)	54.87 ± 12.28	54.91 ± 11.95	54.56 ± 14.90	NA
In-hospital days before LT (mean ± SD)	3.55 ± 11.19	3.54 ± 11.71	3.67 ± 5.14	NA
Total in-hospital days (mean ± SD)	19.95 ± 23.11	18.43 ± 22.87	32.73 ± 21.33	NA
Annual mortality, n (%)	48 (12.8)	44 (13.1)	4 (10.0)	NA
30-day mortality, n (%)	26 (6.9)	24 (7.1)	2 (5.0)	NA
Lost to follow-up, n (%)	27 (7.2)	24 (6.4)	3 (7.5)	NA
Medical history				
Previous liver transplant, n (%)	29 (7.7)	26 (7.7)	3 (7.5)	NA
Acute liver failure, n (%)	20 (5.3)	20 (6.0)	0 (0.0)	NA
Chronic liver failure, n (%)	328 (87.2)	297 (88.4)	31 (77.5)	0.089
Acute chronic liver failure, n (%)	28 (7.4)	19 (5.7)	9 (22.5)	<0.001
Hepatocellular carcinoma, n (%)	89 (23.7)	81 (24.1)	8 (20.0)	NA
Chronic hyponatremia, n (%)	85 (22.6)	71 (21.1)	14 (35.0)	0.075
Previous kidney failure, n (%)	83 (22.1)	73 (21.7)	10 (25.0)	NA
History of PSE, n (%)	175 (46.5)	151 (44.9)	24 (60.0)	NA
Prior epilepsy diagnosis, n (%)	16 (4.3)	9 (2.7)	7 (17.5)	<0.001
Pre-transplant data				
Active antibiotic therapy, n (%)	40 (10.6)	32 (9.5)	8 (20.0)	0.078
Mechanical ventilation, n (%)	20 (5.3)	16 (4.8)	4 (10.0)	NA
Hemodialysis, n (%)	13 (3.5)	10 (3.0)	3 (7.5)	NA
Active PSE, n (%)	120 (31.9)	100 (29.8)	20 (50.0)	0.016
Filtration rate (MDRD, mean ± SD)	82.35 ± 53.27	82.43 ± 53.58	81.74 ± 51.44	NA
MELD sodium (mean ± SD)	22.61 ± 10.24	22.30 ± 10.43	24.86 ± 8.46	NA
MELD (mean ± SD)	19.65 ± 10.34	19.31 ± 10.52	22.10 ± 8.69	NA
Sodium (mean ± SD)	134.72 ± 5.03	134.89 ± 4.93	133.45 ± 5.72	NA
Hemoglobin (mean ± SD)	10.82 ± 2.26	10.84 ± 2.28	10.65 ± 2.10	NA
Potassium (mean ± SD)	4.09 ± 2.17	3.98 ± 0.62	4.96 ± 6.18	NA
INR (mean ± SD)	2.21 ± 1.56	2.24 ± 1.64	2.03 ± 0.75	NA
Creatinine (mean ± SD)	1.41 ± 1.95	1.43 ± 2.03	1.30 ± 1.13	NA
Total bilirubin (mean ± SD)	8.74 ± 10.23	8.18 ± 9.87	12.75 ± 11.92	NA
Albumin (mean ± SD)	2.80 ± 0.68	2.82 ± 0.68	2.68 ± 0.63	NA
Post-surgery sodium (mean ± SD)	139.08 ± 5.06	139.14 ± 4.81	138.55 ± 6.83	NA
Urgent transplant, n (%)	58 (15.4)	49 (14.6)	9 (22.5)	NA
Combined liver-renal transplant, n (%)	34 (9.2)	28 (8.5)	6 (15.0)	NA
Surgical data				
Procedure duration (min, mean ± SD)	403.99 ± 120.30	399.74 ± 116.99	437.03 ± 140.94	NA
Graft cold ischemia time (min, mean ± SD)	435.88 ± 353.17	431.45 ± 366.88	473.79 ± 200.02	0.007
Platelet transfusion, n (%)	100 (26.8)	82 (24.6)	18 (46.2)	NA
Graft complications, n (%)	97 (25.8)	82 (24.4)	15 (37.5)	<0.001
Functional delay/primary failure, n (%)	54 (14.5)	40 (12.0)	14 (35.0)	0.068
Graft rejection, n (%)	33 (8.8)	26 (7.8)	7 (17.9)	NA
Biliary graft complication, n (%)	14 (3.8)	12 (3.6)	2 (5.0)	NA
Graft thrombosis, n (%)	20 (5.4)	17 (5.1)	3 (7.5)	NA
Anastomotic bleeding, n (%)	47 (12.7)	40 (12.1)	7 (17.5)	NA
Subsequent surgeries, n (%)	76 (20.3)	61 (18.3)	15 (37.5)	0.001
New infection after surgery, n (%)	137 (36.5)	112 (33.4)	25 (62.5)	0.068
New renal failure after surgery, n (%)	76 (20.3)	63 (18.8)	13 (32.5)	NA
Shift sodium after surgery (mean ± SD)	-4.27 ± 4.87	-4.16 ± 4.68	-5.10 ± 6.11	NA
Immunotherapy used				
Tacrolimus, n (%)	348 (93.3)	314 (94.0)	34 (87.2)	NA
Mycophenolate, n (%)	256 (68.4)	231 (69.2)	25 (62.5)	NA
Cyclosporine, n (%)	2 (0.5)	1 (0.3)	1 (2.5)	
Methylprednisone, n (%)	358 (96.0)	321 (96.1)	37 (94.9)	

Pre-transplant and intraoperative data

Pre-LT blood tests revealed abnormalities, including low hemoglobin levels (mean = 10.8 g/dL, SD = 2.2), hyponatremia (mean = 134 mEq/L, SD = 5), elevated international normalized ratio (mean = 2.21, SD = 1.56), high total bilirubin levels (mean = 8.74 mg/dL, SD = 10.23), and mild hyperkalemia (mean = 4.09 mmol/L, SD = 2.17). Combined liver and kidney transplants accounted for 9.2% (34) of cases, and 15.4% (58) were performed as emergency procedures. Graft complications occurred in 25.8% (97) of cases, with the most frequent being functional delay (14.5%, 54). Additional complications included graft rejection (8.8%, 33), anastomotic bleeding (12.7%, 47), biliary complications (3.8%, 14), and thrombosis (5.4%, 20). Reoperations were required in 20.3% (76) of patients. The predominant immunosuppressive regimen consisted of methylprednisolone (96%, 358) combined with tacrolimus (93.3%, 348) or mycophenolate (68.4%, 256). Further details are provided in Table 1.

Seizure characteristics

Seizures typically occurred on day seven post-LT (IQR = 5-13). Seizure types were classified as uncertain onset (40%, 16), NCSE (30%, 12), generalized SZ (22.5%, 9), CSE (5%, 2), and focal seizures (2.5%, 1). Most seizure patients (85%, 34) received ASMs, predominantly levetiracetam (75%, 26). Other ASMs included lacosamide (5%, 2) and phenytoin or topiramate (5%, 2). No cases of super-refractory status epilepticus were observed. Acute metabolic triggers were identified in 35% (14) of seizure patients, including severe hypocalcemia (8) and significant sodium shifts (>10 mEq/L in 24 hours, n = 2). Structural brain lesions, identified in 18% (7) of seizure patients, included pontine/extrapontine myelinolysis (3), central aspergillosis (1), subacute putaminal hematoma (1), pyramidal Wallerian degeneration (1), and acute pontine ischemia (1). At three years post-LT, 13% (5) of seizure patients experienced at least one recurrence, although no cases of NCSE or CSE were reported.

Comparative analysis

Patients with seizures were more likely to have a prior epilepsy diagnosis (17.5% vs. 2.7%, p < 0.001), acute-on-chronic liver failure (22.5% vs. 5.7%, p < 0.001), or active PSE pre-LT (50% vs. 29.8%, p = 0.016). They also had higher rates of platelet transfusion (46.2% vs. 24.6%, p = 0.007), primary graft failure (35% vs. 12%, p < 0.001), and post-LT infections (62.5% vs. 33.4%, p = 0.001). These findings highlight key risk factors associated with seizures after LT.

Predictive modeling and risk score

A predictive model for seizures after LT was developed and tested using variables selected for their clinical relevance and statistical performance. These variables included age at transplantation, history of PSE, prior epilepsy diagnosis, pre-LT hemoglobin levels, procedure duration, graft cold ischemia time, and intraoperative blood transfusion. The model achieved a specificity of 93%, sensitivity of 42%, AUC of 0.82, kappa of 0.35, and accuracy of 0.87 (95% confidence interval (CI) = 0.80-0.93), demonstrating good predictive capacity.

Based on the predictive model, a risk score was developed, as seen in Table 2, to stratify patients into risk categories (moderate, high, and very high) based on their likelihood of developing seizures after LT, as seen in Table 3. The score demonstrated strong performance metrics, including specificity of 100%, sensitivity of 69%, AUC of 0.90, kappa of 0.32, and Accuracy of 0.72 (95% CI = 0.63-0.80).

**Table 2:** Scores for each predictive variable. *: before transplantation. PSE: portosystemic encephalopathy; LT: liver transplantation

Predictive variables scores	Classification	Score
Procedure duration (hours)	0–6	0
	6.1–7	5
	7.1–8	15
	8.1–10	5
	>10	0
Graft cold ischemia time (hours)	0–5	0
	5.1–6	5
	6.1–7	10
	>7	15
Intraoperative blood transfusion	No	0
	Yes	15
Age at transplantation (years)	18–45	0
	45.1–50	5
	50.1–55	10
	55.1–60	15
	>60	20
History of PSE*	No	0
	Yes	15
Prior epilepsy diagnosis*	No	0
	Yes	10
Pre-LT hemoglobin levels (g/dL)*	0–8	15
	8.1–9	10
	9.1–10	5
	>10	0

**Table 3:** Predictive score risk stratification for seizure after liver transplantation.

Total score	Risk category	Seizure risk seizure risk	Recommendations
0–34	Low risk	25%	Routine follow-up
35–49	Moderate risk	50%	Increased monitoring
50–59	High risk	75%	Intensive monitoring
60–100	Very high risk	100%	Immediate intervention should be considered

## Discussion

Our study identified a 10.6% incidence of seizures among 376 consecutively analyzed patients undergoing LT, aligning with the broad range of seizure rates (3-42%) reported by previous research [4,6,7,11,16,19-24]. We also developed a predictive risk score, grounded in in-hospital variables, that demonstrated strong performance in anticipating post-LT seizures. While numerous publications have evaluated clinical factors linked to neurological complications after LT, relatively few have specifically addressed seizure incidence and characterization, underscoring our study’s relevance in deepening the understanding of seizure dynamics in LT recipients.

The 10.6% seizure incidence and the observed spectrum of seizure types are consistent with prior investigations. For example, a retrospective study of 142 LT patients reported a 15.7% seizure incidence, with 10.5% of cases arising in the immediate postoperative period; in that cohort, 13% of seizures were classified as CSE, while the remainder showed generalized onset [14]. Another retrospective series of 367 patients noted a 4.6% seizure incidence post-LT, with a 52% recurrence rate on the same day [19]. Broader assessments of neurological complications, including one with 288 LT patients, reported a 7% seizure incidence, further corroborating our findings [6].

Although generalized-onset seizures, often linked to immunosuppressant neurotoxicity, are commonly observed in LT populations, most seizures in our study were categorized as uncertain onset or NCSE. Two main factors may account for this discrepancy: (1) the 2017 ILAE classification, which introduced the uncertain-onset category, and (2) difficulties in documenting brief or infrequent events within DMRs. Nevertheless, the frequency of triggers such as structural lesions (13%) and metabolic disturbances (20-33%) remains consistent with previous literature [14].

Evidence on seizure management in LT recipients remains limited, as no randomized controlled trials have established the efficacy of specific ASMs in this setting. At our center, levetiracetam was the primary ASM due to its minimal interaction with immunosuppressive therapies, followed by lacosamide. Other studies similarly identify levetiracetam as the most frequently used ASM (52.2%), with phenytoin ranking second (17.4%) [14].

Pre-LT risk factors are critical for predicting seizures during the immediate postoperative period. One regional study identified severe ascites, shifts in serum sodium, and hypomagnesemia as relevant predictors for neurological complications (including seizures), yielding an ROC curve of 0.71 [4]. A U.S.-based investigation cited pre-LT infections as the sole significant predictor in multivariate analysis, predominantly associated with PSE and tremors, rather than seizures specifically [5]. To our knowledge, few studies have focused on seizure-specific risk factors in the LT population.

Contrary to certain reports indicating higher mortality among LT recipients with seizures [17], we observed no significant difference in 30-day or one-year mortality between seizure and non-seizure groups. Most seizures in our cohort were self-limited and not associated with major structural lesions or severe metabolic aberrations. We hypothesize that this, combined with prompt recognition and early administration of ASMs, contributed to favorable outcomes and shorter hospital stays.

Several limitations of this study merit discussion. The retrospective design could lead to incomplete data capture and reporting biases, despite attempts to mitigate these through sensitivity analyses and the exclusion of incomplete cases. As the predictive model was developed in a single tertiary center, its generalizability may be limited, calling for multicenter validation. Subgroup analyses were constrained by small sample sizes, limiting statistical power. Future prospective, multicenter research could standardize data collection, expand sample sizes, and refine the model’s applicability across diverse settings, ultimately enhancing post-LT interventions.

Despite these caveats, our investigation comprehensively evaluates perioperative seizure risk in LT recipients and presents a predictive model with robust performance metrics. Strengths include its internal validation; detailed profiling of clinical, laboratory, and perioperative variables; and the defined seven-day pre-LT to 30-day post-LT window capturing pivotal preoperative and early postoperative factors. From a clinical standpoint, the model could be readily adopted into perioperative workflows, enabling teams to identify high-risk patients, start neurological surveillance, address metabolic derangements, or consider prophylactic ASMs. Nonetheless, barriers to its widespread implementation remain, including the need for comprehensive data capture, multidisciplinary collaboration, and tailored protocol adaptations.

## Conclusions

Seizures remain a notable complication of liver LT, as demonstrated by a 10.6% incidence among 376 consecutively analyzed patients in our study. Using a random forest approach, we developed and internally validated a predictive model and corresponding risk score, incorporating clinically relevant factors (age at transplantation, history of PSE pre-LT, prior epilepsy diagnosis, pre-LT hemoglobin levels, procedure duration, graft cold ischemia time, and intraoperative blood transfusion). This model exhibited strong performance in identifying patients at increased risk of post-LT seizures.

By highlighting key risk factors, our findings support more tailored perioperative management and proactive seizure mitigation strategies. Nevertheless, prospective multicenter validation is necessary to confirm the model’s generalizability and refine preventive measures, thereby optimizing neurological outcomes in this patient population.
